# Clinical and biochemical factors associated with amygdalar metabolic activity

**DOI:** 10.1038/s41514-025-00194-4

**Published:** 2025-01-25

**Authors:** Atsuko Tahara, Nobuhiro Tahara, Akihiro Honda, Sachiyo Igata, Munehisa Bekki, Shoko Maeda-Ogata, Yuki Koga, Ruiko Nonaka, Kenta Murotani, Shuichi Tanoue, Sho-ichi Yamagishi, Yoshihiro Fukumoto

**Affiliations:** 1 Division of Cardiovascular Medicine, Department of Medicine, Kurume University School of Medicine, 67 Asahi-machi, Kurume, Japan; 2 Biostatistics Center, Kurume University, Kurume, Japan; 3 School of Medical Technology, Kurume University, Kurume, Japan; 4 Department of Radiology, Kurume University School of Medicine, Kurume, Japan; 5 Division of Diabetes, Metabolism, and Endocrinology, Department of Medicine, Showa University Graduate School of Medicine, Tokyo, Japan

**Keywords:** Cardiovascular diseases, Metabolic disorders, Biomarkers

## Abstract

We investigated clinical factors and biochemical markers associated with amygdalar metabolic activity evaluated by [^18^F]-fluorodeoxyglucose-positron emission tomography (FDG-PET) in 346 subjects without a history of malignant neoplasms. Univariate regression analysis revealed significant relationships between amygdalar metabolic activity and fasting plasma glucose (FPG), glycated hemoglobin, coronary artery disease (CAD) history, aspirin use, oral hypoglycemic agents (OHAs) use, and asymmetric dimethylarginine (ADMA). In multiple stepwise regression analysis, FPG and CAD history were independently associated with amygdalar metabolic activity. Moreover, in 36 patients with type 2 diabetes mellitus (T2DM), additional OHAs treatment significantly improved glycemic and metabolic parameters, and decreased ADMA concentrations. Baseline and Δpigment epithelium-derived factor (PEDF), a marker of insulin resistance, was a significant associate with Δamygdalar metabolic activity. Our study demonstrates that FPG and CAD history were independently associated with amygdalar metabolic activity in subjects without a history of malignant neoplasms. In T2DM patients, PEDF might regulate amygdala metabolic activity.

## Introduction

Our lives have become comfortable along with improvement of convenience for living, development of information society, and diversity of life style^1^. On the other hand, we unconsciously receive environmental, chemical, biological, social, mental, and economic stress under the living conditions^1^. Especially, psychosocial stress has been increasing in modern life. These stressors are associated with adverse health behaviors, such as excessive caloric consumption and physical inactivity, which could lead to aging-related metabolic disorders, including insulin resistance, obesity, type 2 diabetes mellitus (T2DM), and eventually to cardiovascular disease (CVD)^2^. Amygdala is a key component of neurobiological network that regulates physiologic and behavioral changes in response to stress and emotion^3^. Psychological stress is known to prompt the amygdala’s projections to the brainstem, thereby triggering the activation of sympathetic nervous system, and hypothalamic-pituitary-adrenal (HPA) axis, which contribute to increases in circulating catecholamines and glucocorticoids, respectively, thus resulting in hyperglycemia^2^.

The prevalence and incidence of T2DM are dramatically increasing due to an aging of the world population^4^. T2DM patients are at high risk of CVD^4^. Furthermore, aging is also strongly associated with the development and progression of atherosclerotic CVD^5,6^. In addition, large prospective studies have shown the association of anxiety and depression with CVD, thus suggesting that chronic psychological stress may influence the prevalence and severity of CVD^7–9^. These observations suggest that evaluation and control of psychological stress may be a novel therapeutic strategy to reduce the risk of T2DM and CVD, especially in world’s aging population. However, it is hard to objectively quantify the degree of psychological stress. The hurdle to measure the degree of stress prohibits us to elucidate the pathophysiology of its relationship with CVD. Positron emission tomography (PET) combined with computed tomography (CT) is a reproducible imaging tool for quantitatively evaluating the activity of neural circuits in the brain^10^. 18F-fluorodeoxyglucose (FDG)-PET/CT has been used as a non-invasive imaging modality to assess the brain activity by quantifying glucose metabolism^11^. Given that amygdala is a main component of stress response, amygdalar metabolic activity evaluated by FDG-PET/CT could be used as a biomarker of stress levels in humans. Indeed, an increased amygdalar metabolic activity evaluated by FDG-PET/CT was identified as an independent risk predictor of major adverse cardiac events (MACE) in subjects with and without coronary artery disease (CAD)^12,13^. Furthermore, FDG-PET/CT imaging enables quantification of neurobiological activity enhanced by environmental noise and socioeconomic stressors, which are associated with the incidence and prevalence of CVD^14–16^. Elucidating and targeting the residual cardiovascular risks in primary and secondary prevention may allow a development of novel therapeutic strategies. However, little is known about the factors related to amygdalar metabolic activity. Here, we measured the amygdalar metabolic activity using FDG-PET/CT and determined which clinical and biochemical factors were associated with amygdalar metabolic activity in patients who underwent a risk-screening test for CVD.

## Results

### Clinical characteristics of subjects in study design 1

**Fig. 1:**
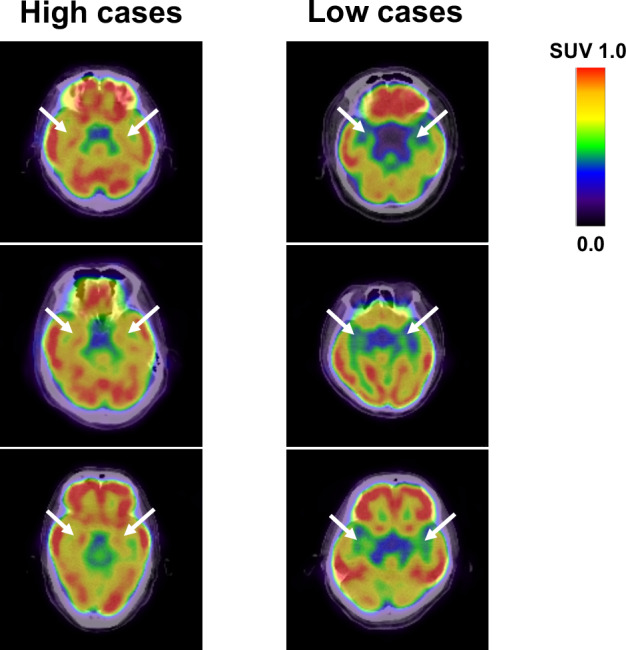
Representative FDG-PET/CT images of 6 cases. Axial views of the brain FDG/PET imaging in 3 cases with high amygdalar metabolic activity (left panels) and 3 with low activity (right panels). SUV standardized uptake value.

Clinical characteristics of subjects in the study design 1 are presented in Table 1. The study involved 346 subjects (232 males and 114 females) with a mean age of 63.2 ± 9.2. None of the subjects had a history of depression or anxiety. Sixty-three subjects were active smokers. Two hundred twenty subjects had essential hypertension. Of these, 137 patients received anti-hypertensive agents. Ninety subjects were T2DM, in whom 49 patients received oral hypoglycemic agents (OHAs). Eighty-nine subjects had been treated with statins, 52 with aspirin, and 26 with sleeping pills. There were 11 patients with cerebrovascular disease and 33 with CAD. Hepatic and bile enzymes, lipid profile, and renal function presented normal values. Serum levels of high-sensitivity CRP (hsCRP), and asymmetric dimethylarginine (ADMA) were 0.49 (0.25–0.99) mg/L and 0.47 (0.42–0.53) nmoL/mL, respectively. FDG activity within the amygdalar was 0.72 ± 0.06. There were no significant differences in age, blood pressure, low-density lipoprotein (LDL) cholesterol, fasting immunoreactive insulin (IRI), HbA_1c_, hsCRP, ADMA, pigment epithelium-derived factor (PEDF; a novel marker of insulin resistance), and amygdalar FDG activity between males and females. Body mass index, waist circumference, liver transaminases, γ-glutamyl transferase (γ-GTP), triglycerides, fasting plasma glucose (FPG), uric acid, areas of visceral adipose tissue (VAT), and subcutaneous adipose tissue (SAT), and percentage of active smoker in males were significantly higher than those in females. On the other hand, heart rate, high-density lipoprotein (HDL) cholesterol, estimated glomerular filtration rate (eGFR), and adiponectin were higher in females. Figure 1 shows representative FDG-PET/CT images of 6 cases; 3 with high metabolic activity in the amygdala (left panels) and 3 with low activity (right panels).

**Table 1:** Clinical variables of subjects

Clinical variables	All	Male	Female	P value
Number	346	232	114	
Age, years	63.2 ± 9.2	63.2 ± 9.0	63.1 ± 9.8	0.960
Body mass index, kg/m^2^	23.9 ± 3.3	24.2 ± 2.9	23.2 ± 3.9	**0.014**
Waist circumference, cm	86.8 ± 9.8	89.2 ± 7.6	81.8 ± 11.4	**<0.001**
Heart rate, beats*/minute	64.1 ± 10.0	63.2 ± 10.1	66.0 ± 9.5	**0.013**
Systolic blood pressure*, mmHg	135.3 ± 18.2	134.7 ± 17.1	136.4 ± 20.2	0.425
Diastolic blood pressure*, mmHg	80.3 ± 11.4	80.9 ± 11.2	79.0 ± 11.6	0.147
Aspartate transaminase*, U/L	23.0 (19.0–28.0)	24.0 (20.0–29.0)	21.0 (18.8–24.3)	**0.025**
Alanine transaminase*, U/L	20.0 (16.0–28.0)	22.0 (17.0–32.8)	18.0 (13.0–23.3)	**<0.001**
γ-glutamyl transferase*, U/L	30.0 (20.0–53.3)	36.0 (23.0–67.0)	21.5 (15.0–30.0)	**<0.001**
LDL cholesterol, mg/dL	123.6 ± 29.4	122.3 ± 29.6	126.2 ± 28.8	0.253
HDL cholesterol, mg/dL	57.1 ± 14.1	53.8 ± 12.7	63.7 ± 14.5	**<0.001**
Triglycerides*, mg/dL	102.0 (73.8–146.0)	114.0 (78.3–156.3)	91.5 (62.0–126.5)	**<0.001**
Fasting plasma glucose*, mg/dL	101.0 (93.0–114.3)	103.0 (96.0–118.8)	96.0 (89.8–105.0)	**0.001**
Fasting immunoreactive insulin*, mU/mL	5.20 (3.40–7.93)	5.20 (3.43–7.98)	5.15 (3.38–7.93)	0.530
Glycated hemoglobin, %	6.05 ± 0.75	6.09 ± 0.81	5.96 ± 0.60	0.140
Estimated glomerular filtration rate, mL/min	77.1 ± 18.0	75.1 ± 15.1	81.2 ± 22.3	**0.003**
Uric acid, mg/dL	5.72 ± 1.39	6.07 ± 1.30	5.02 ± 1.29	**<0.001**
High-sensitivity CRP*, mg/L	0.49 (0.25–0.99)	0.49 (0.27–0.93)	0.50 (0.19–1.42)	0.623
ADMA*, nmoL/mL	0.47 (0.42–0.53)	0.47 (0.42–0.54)	0.47 (0.42–0.50)	0.188
PEDF*, mg/mL	12.25 (9.02–16.00)	13.70 (9.74–17.50)	10.10 (7.92–13.65)	**<0.001**
Visceral adipose tissue area, cm^2^	118.8 ± 45.6	118.8 ± 45.7	90.0 ± 36.0	**<0.001**
Subcutaneous adipose tissue area, cm^2^	127.8 ± 41.1	127.8 ± 41.2	139.2 ± 49.7	**0.004**
Amygdalar metabolic activity	0.72 ± 0.06	0.72 ± 0.07	0.71 ± 0.05	0.177
Active smoker, n (%)	63 (18.2)	51 (22.2)	14 (4.0)	**0.030**
Cerebrovascular disease, n (%)	11 (3.2)	9 (3.9)	2 (0.6)	0.464
Coronary artery disease, n (%)	33 (9.5)	28 (12.1)	5 (1.4)	**0.022**
Drugs, n (%)				
Aspirin	52 (15.0)	44 (19.0)	8 (7.0)	**0.004**
Statins	89 (25.7)	60 (25.9)	29 (25.4)	0.933
Anti-hypertensive agents	137 (39.6)	103 (44.4)	34 (29.8)	**0.009**
Oral hypoglycemic agents	49 (14.2)	40 (17.2)	9 (7.9)	**0.019**
Sleeping pills	26 (7.5)	15 (6.5)	11 (9.6)	0.291

Data are presented as number (n) (%), mean ± SD, or *median (interquartile range).

*LDL* low-density lipoprotein, *HDL* high-density protein, *CRP* C-reactive protein, *ADMA* asymmetric dimethyl arginine, *PEDF* pigment epithelium-derived factor.

Bold indicates the statistical significance of values between male and female.

### Association between amygdalar metabolic activity and clinical variables

**Fig. 2:**
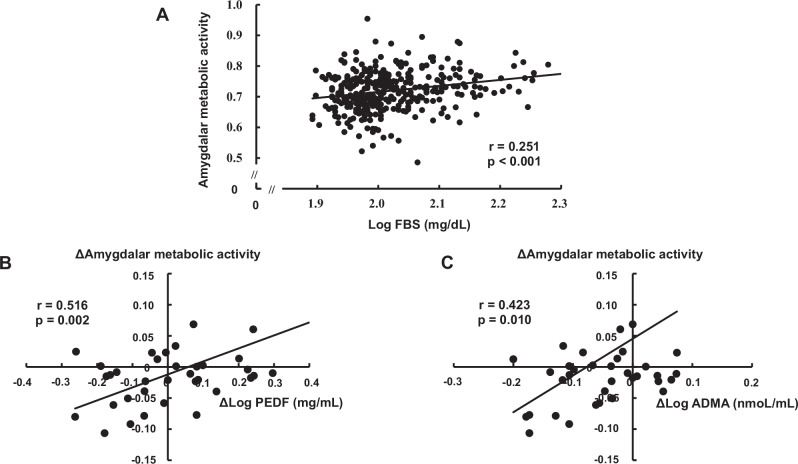
Plots of significant correlations. **A** Correlation of FPG with amygdalar FDG activity in 346 subjects. **B** Correlation of DPEDF with Damygdalar metabolic activity in T2DM patients. **C** Correlation of DADMA with Damygdalar metabolic activity in T2DM patients.

Table 2 shows the results of univariate and multivariate regression analyses for determinants of amygdalar FDG activity. As shown in Table 2 and Fig. 2A, FPG (p < 0.001), HbA_1c_ (p < 0.001), ADMA (p = 0.034), CAD history (p < 0.001), aspirin use (p = 0.013), and OHAs use (p < 0.001) were significantly correlated with amygdalar FDG activity in univariate analysis. The least absolute shrinkage and selection operator (LASSO) demonstrated that FPG (p = 0.010), HbA_1c_ (p < 0.001), CAD history (p = 0.007), and OHAs use (p = 0.002) were significant variables. Because these significant variables could be closely correlated with each other, we performed multiple stepwise regression analysis to determine independent associates of amygdalar FDG activity. FPG (p < 0.001) and CAD history (p = 0.010) were independently associated with amygdalar FDG activity (R^2^ = 0.076). The study design 1 included 90 patients with T2DM and 256 non-DM subjects, whom clinical characteristics are described in Supplementary Table 1. No significant differences were observed between the two groups in body mass index, waist circumference, heart rate, hepatic and bile enzymes, LDL cholesterol, triglycerides, uric acid, PEDF, areas of VAT, and SAT, smoking status, and sleep pills use. Compared to the non-DM group, the T2DM group was older, and male predominance, and had higher hsCRP, ADMA, use of medicines including aspirin, statins, hypertensive agents and OHAs, and amygdalar FDG activity (0.75 ± 0.06 vs 0.71 ± 0.06, p < 0.001). On the other hand, eGFR, and HDL cholesterol in the T2DM group were lower than those in the non-DM group.

**Table 2:** Associations between clinical variables and amygdalar metabolic activity at baseline

Parameters	Univariate		Multivariate	
	β	P value	β	P value
Gender^a^	−0.073	0.177	-	-
Age	0.017	0.760	-	-
Body weight	0.030	0.576	-	-
Waist circumference	0.027	0.616	-	-
Body mass index	0.036	0.504	-	-
Heart rate	0.082	0.128	-	-
Systolic blood pressure	0.023	0.666	-	-
Diastolic blood pressure	0.016	0.773	-	-
Aspartate transaminase^b^	0.043	0.425	-	-
Alanine transaminase^b^	0.069	0.200	-	-
γ-glutamyl transferase^b^	0.101	0.061	-	-
LDL cholesterol	0.000	0.995	-	-
HDL cholesterol	−0.089	0.097	-	-
Triglycerides^b^	0.063	0.244	-	-
Fasting plasma glucose^b^	0.251	**<0.001**	0.204	**<0.001**
Fasting immunoreactive insulin^b^	0.094	0.080	-	-
Glycated hemoglobin	0.217	**<0.001**	0.048	0.573
Estimated glomerular filtration rate	−0.003	0.954	-	-
Uric acid	0.092	0.087	-	-
High-sensitivity CRP^b^	0.062	0.273	-	-
ADMA^b^	0.114	**0.034**	-	-
PEDF^b^	0.027	0.621	-	-
Visceral adipose tissue area	0.031	0.630	-	-
Subcutaneous adipose tissue area	0.028	0.662	-	-
Current smoking^a^	−0.028	0.603	-	-
Coronary artery disease^a^	0.211	**<0.001**	0.142	**0.010**
Cerebrovascular injury^a^	0.076	0.158	-	-
Aspirin^a^	0.134	**0.013**	-	-
Statins^a^	0.091	0.092	-	-
Medication for hypertension^a^	0.019	0.725	-	-
Oral hypoglycemic agents^a^	0.186	**<0.001**	0.061	0.308
Sleeping pills^a^	−0.007	0.900	-	-
R^2^			0.076	

β, regression coefficients.

Abbreviations as in Table 1.

^a^Men = 0, Women = 1 or No = 0, Yes = 1.

^b^Log-transformed value was used.

Bold indicates the statistical significance.

### Effects of OHAs on amygdalar metabolic activity

**Fig. 3:**
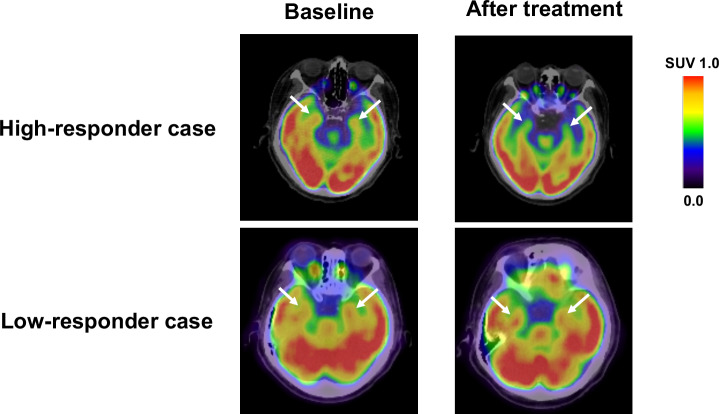
FDG-PET/CT images at baseline and after 16-week treatment with OHAs. FDG-PET/CT images of high-responder and low-responder case. After 16-week add-on OHA therapy, there were some patients, whose amygdala metabolic activity was drastically decreased in response to the additional treatment of OHAs.

In the study design 2, 36 patients with T2DM received additional pioglitazone 15–30 mg daily (mean titrated daily dose 17.6 ± 5.7 mg) or glimepiride 0.5–4 mg daily (mean titrated daily dose 1.3 ± 1.1 mg) for 16 weeks. Treatments of OHAs were well tolerated, and there were no adverse effects of drugs, such as severe hypoglycemia, or heart failure. Supplementary Table 2 demonstrates the clinical variables at baseline and after the 16-week treatment with OHAs. The additional OHAs therapy significantly reduced FPG and HbA1c levels. After the OHAs treatments, triglycerides, and ADMA levels were significantly decreased, while body weight, waist circumference, and HDL cholesterol were significantly increased. There were no significant changes in heart rate, blood pressure, hepatic, and bile enzymes, fasting IRI, hsCRP, uric acid, eGFR, PEDF, or areas of VAT and SAT after 16-week add-on therapy. Although mean values of amygdalar metabolic activity did not change after 16-week add-on OHA therapy, there were some patients, whose amygdala metabolic activity was drastically decreased in response to the additional treatment of OHAs (Fig. 3).

### Association of baseline clinical variables and ∆clinical variables with ∆amygdalar metabolic activity

Regression analysis revealed that PEDF level at baseline was a sole and significant associate with Δamygdalar metabolic activity in 36 patients with T2DM (Supplementary Table 3). Next, we examined the association of ∆clinical variables with Δamygdalar metabolic activity in 36 T2DM patients. As shown in Table 3, ∆γ-GTP, ΔhsCRP, ΔADMA, and ΔPEDF were positively correlated with Δamygdalar metabolic activity in univariate analysis, and then all of them were identified as significant variables by LASSO regression analysis. Multiple stepwise regression analysis revealed the statistically significant and independent association of ΔPEDF with Δamygdalar metabolic activity (R^2^ = 0.244) (Fig. 2B). Because ΔADMA and ΔPEDF were closely correlated with each other (r = 0.475, p = 0.004), a second model of multiple stepwise regression analysis was performed to determine the independent associates of Δamygdalar metabolic activity other than ΔPEDF. As shown in Supplementary Table 4, ΔADMA remained significant and was independently associated with Δamygdalar metabolic activity (R^2^ = 0.155) (Fig. 2C).

**Table 3:** Association of Δclinical variables with Δamygdalar metabolic activity

Parameters	Univariate		Multivariate	
	β	P value	β	P value
ΔBody weight	−0.174	0.309	-	-
ΔWaist circumference	−0.226	0.185	-	-
ΔHeart rate	−0.081	0.638	-	-
ΔSystolic blood pressure	0.153	0.372	-	-
ΔDiastolic blood pressure	−0.086	0.619	-	-
ΔAspartate transaminase^a^	0.142	0.408	-	-
ΔAlanine transaminase^a^	0.323	0.055	-	-
Δγ-glutamyl transferase^a^	0.372	**0.025**	0.194	0.227
ΔLDL cholesterol	0.032	0.853	-	-
ΔHDL cholesterol	−0.186	0.277	-	-
ΔTriglycerides^a^	−0.058	0.735	-	-
ΔFasting plasma glucose^a^	0.206	0.229	-	-
ΔFasting immunoreactive insulin^a^	−0.099	0.565	-	-
ΔGlycated hemoglobin	0.060	0.728	-	-
ΔUric acid	0.277	0.102	-	-
ΔHigh-sensitivity CRP^a^	0.332	**0.048**	0.155	0.343
ΔADMA^a^	0.423	**0.010**	0.217	0.206
ΔPEDF^a^	0.516	**0.002**	0.516	**0.002**
ΔVisceral adipose tissue area	0.001	0.993	-	-
ΔSubcutaneous adipose tissue area	−0.226	0.185	-	-
R^2^			0.244	

β: regression coefficients.

Abbreviations as in Table 1.

^a^Log-transformed value was used.

Bold indicates the statistical significance.

### ΔPEDF and ΔADMA stratified by median value of Δamygdalar metabolic activity

When 36 T2DM patients were divided into two groups by median value of ∆amygdalar metabolic activity (−0.013) [low ∆amygdalar metabolic activity group with ∆amygdalar metabolic activity ≤ −0.013 (high responders to OHAs treatment) versus high ∆amygdalar metabolic activity group with ∆amygdalar metabolic activity > −0.013 (low responders to OHAs treatment)], amygdalar metabolic activity at baseline in the high-responder group was significantly higher than that of low-responder group. Although FPG and HbA_1c_ levels were comparably reduced in the two groups by add-on OHAs therapy, ALT, γ-GTP, hsCRP, and ADMA values were significantly decreased and HDL cholesterol was significantly increased in the high-responder group, but not in the low-responder group (Supplementary Table 5).

## Discussion

The major findings of our study are as follows; (1) while FPG, HbA_1c_, CAD history, aspirin use, OHAs use, and ADMA were significantly correlated with amygdalar metabolic activity evaluated by FDG-PET/CT in subjects without a history of malignant neoplasms in univariate analysis, FPG and CAD history were independently associated with amygdalar metabolic activity in multivariate analysis, (2) amygdalar metabolic activity in T2DM patients was higher than that in non-DM patients, (3) there were some T2DM patients with considerably decreased amygdalar metabolic activity in response to additional OHAs therapy, (4) Baseline PEDF level was inversely and independently associated with Δamygdalar metabolic activity in T2DM patients, (5) ΔPEDF was a sole factor which was positively and independently associated with Δamygdalar metabolic activity in T2DM patients, whereas Δγ-GTP, ΔhsCRP, ΔADMA and ΔPEDF were significantly correlated with Δamygdalar metabolic activity in univariate analysis, and (6) when we performed multivariate analysis by excluding the data of ΔPEDF, only ΔADMA was independently correlated with Δamygdalar metabolic activity. The present study demonstrates that amygdalar metabolic activity is increased under fasting hyperglycemic condition or in patients with a history of CAD, and reduction of amygdalar metabolic activity is independently associated with baseline serum PEDF and ΔPEDF in patients with T2DM.

A growing body of epidemiological evidence has suggested that psychosocial stress adversely affects glycemic control among individuals with pre-existing diabetes and thereby could contribute to the development of diabetes^17,18^. Excess caloric intake, physical inactivity, and activation of sympathetic nervous system and HPA axis under psychological stress conditions may deteriorate glycemic control in these patients^2^. On the other hand, diabetes patients are at increased risk for anxiety, depression, and emotional distress, and fasting hyperglycemia is associated with these mental disorders^19,20^. In this study, FPG and CAD history were independently associated with amygdalar metabolic activity in subjects without a history of malignant neoplasms. Given the present findings that amygdalar metabolic activity, which is also known to be increased in affective and neurotic disorders^21,22^, was significantly higher in T2DM patients compared to non-DM patients, FPG, and psychological stress evaluated by amygdalar metabolic activity may have a bidirectional interaction, which could promote the development of CAD, thus explaining why FPG and CAD history were independent associates of amygdalar metabolic activity in the study 1. Stress-related amygdalar metabolic activity evaluated by FDG-PET/CT has been shown to be associated with an increased risk of CVD events partly through neuroimmune pathways triggered by stress perception via the activation of the sympathetic nervous system and the HPA axis^12–16^. In addition, coronary artery inflammation assessed by fat attenuation index was significantly correlated with amygdalar metabolic activity in CAD patients^13^. These observations further support our speculation.

The study 1 was a cross-sectional design, which did not elucidate the causal relationship between FPG levels and amygdalar metabolic activity. Therefore, we investigated whether and how add-on OHAs therapy for 36 patients with T2DM could affect amygdalar metabolic activity in the study design 2. As a result, amygdalar metabolic activity remained unchanged after 16-week add-on OHAs therapy. However, there were some T2DM patients with considerably reduced amygdala metabolic activity in response to the additional treatment. Thus, we examined the association of baseline and ∆clinical variables with Δamygdalar metabolic activity. We found in the study 2 that baseline PEDF level and ΔPEDF were independent associates with Δamygdalar metabolic activity in 36 patients with T2DM. PEDF, a glycoprotein that belongs to the superfamily of serine protease inhibitors, was first purified from the conditioned media of human retinal pigment epithelial cells as a factor which possesses potent neuronal differentiating activity^23^. Since then, PEDF has been found to have anti-inflammatory, anti-oxidative, and anti-thrombogenic properties in both cell culture and animal models^24,25^, and its serum levels could be a novel biomarker of insulin resistance and central obesity^26,27^. Indeed, there was a significant correlation between serum levels of PEDF and metabolic risk factors, including waist-to-hip ratio, waist circumference, homeostasis model of assessment of insulin resistance (HOMA-IR) in patients with the metabolic syndrome or T2DM^26,27^. Furthermore, circulating PEDF values were increased in proportion to the number of components of the metabolic syndrome in a general population, and could predict the development of metabolic syndrome^26^. Therefore, our present study suggests that baseline PEDF level is a more sensitive biomarker of insulin resistance than other well-known parameters of insulin resistance and/or central obesity, such as fasting IRI, waist circumference, HOMA-IR, or VAT area, and could predict which T2DM patients’ amygdalar metabolic activity was effectively decreased by add-on OHAs therapy. In T2DM patients who had already treated with OHAs, fasting IRI and/or HOMA-IR index could not necessarily be markers of insulin resistance, thus further supporting the clinical relevance of measuring baseline serum levels of PEDF in identifying T2DM patients who may benefit most from additional OHAs therapy.

In the present study, we found that ΔPEDF was a sole independent associate of Δamygdalar metabolic activity. Given that PEDF alleviates depressive-like behaviors and works as an antidepressant in animal models^28^, our present findings might indicate that PEDF could affect psychological stress evaluated by amygdalar metabolic activity. In other words, serum PEDF levels might be elevated in response to psychological stress as a counter-system. We have previously shown that although circulating PEDF levels are a marker of insulin resistance in both animal models and humans^26,29^, PEDF addition improves hepatic insulin resistance in vitro and ameliorates metabolic derangements in an animal model of T2DM with obesity and insulin resistance^30,31^, thus suggesting that serum PEDF levels are increased as a counter-system against insulin resistance as well. Serum PEDF levels are also a biomarker of atherosclerosis, which are independently associated with vascular inflammation and intima-media thickness of carotid artery in humans^32^. Therefore, although amygdala receives blood supply from the anterior and posterior cerebral circulations, circulating PEDF levels may be a marker that could reflect amygdalar metabolic activity, which may partly explain the association of atherosclerotic risk factors, such as FPG and a history of CAD with amygdalar metabolic activity. However, in the study 1, serum levels of PEDF were not significantly correlated with amygdalar metabolic activity. Accordingly, circulating PEDF levels may a marker to predict the response of amygdalar metabolic activity to additional OHAs therapy in T2DM. In any case, further longitudinal interventional studies are needed to clarify whether and how circulating PEDF could regulate amygdalar metabolic activity.

Plasma concentrations of ADMA, an endogenous inhibitor of nitric oxide (NO) synthase showed a significant correlation with amygdalar metabolic activity in the study design 1 and significantly decreased after additional OHAs therapy in the study design 2. Since ADMA and PEDF was highly correlated with each other^33^, we performed a second model of multiple stepwise regression analysis by excluding the data of ΔPEDF and found that ΔADMA was a sole correlate of Δamygdalar metabolic activity in T2DM patients. There is accumulating evidence that NO is a crucial inter-neuronal messenger in the brain^34^, and NO synthase-expressing neurons exist in the brain^35^. Intravenous administration of ADMA has been reported to cause sympathetic nervous system activation in conscious rats^36^. In addition, plasma concentration of ADMA was associated with sympathetic nervous system activity in obesity patients with T2DM^37^, while NO is known to play an autonomic regulatory role by decreasing sympathetic nerve output^38^. NO-producing neurons were activated during restraint stress in animals experiencing increased levels of sympathetic activity^39^. These observations are consistent with our hypothesis. Given the pathological role of ADMA in atherosclerotic CVD and glucose metabolism^40^, ADMA may play a role in increased amygdalar metabolic activity in T2DM, and could be a factor that explains the link of FPG and a history of CAD to amygdalar metabolic activity.

Our study has several limitations for interpreting the results. First, our study represents a single-center experience, which limits the generalizability of our findings by its selection bias. Second, the sample size and various co-medications may limit and confound the present results, especially for the power of the univariate and multivariate linear regression analysis. Third, questionnaires for the assessment of environmental and socioeconomic stressors were not performed in the present study. Although several studies have shown that increased metabolic activity in the amygdala is closely associated with psychological stress^7–11^, amygdalar metabolic activity is not limited to the psychological stress response alone. Prospective studies are required to confirm that amygdalar metabolic activity is directly related to psychological stress. Fourth, factors associated with amygdalar metabolic activity could be different depending on the regulatory region to be corrected. Also, we could not evaluate the brain FDG activity in other regions related to neural circuits because of limited imaging resolution on FDG-PET/CT analysis. The masks used to evaluate the activity could not be presented in the figures, because the amygdalar metabolic activity was automatically measured by FineSRT. Development of novel PET tracers and novel image analysis tools would provide more specific information for detecting the stress-related neural activity in humans. Fifth, any biomarkers of sympathetic nervous system and HPA axis activity were not measured in our study. We cannot exclude the possibility that unmeasured confounders could affect the present results. Sixth, the study design 2 was open-labeled. The results of this study did not contradict the hypothesis that psychological stress causes increased amygdala activity and consequently exacerbates lifestyle-related diseases, such as diabetes. However, it did not mean that our results provided strong evidence to positively support it. A blinded randomized interventional study with large populations is warranted to confirm our findings. Further longitudinal study is needed to examine whether reduction of amygdalar metabolic activity could indeed contribute to the risk reduction of CVD.

In conclusion, amygdalar metabolic activity is increased under hyperglycemic conditions, and associated with a history of CAD. Our present study indicates that amygdalar metabolic activity in FDG-PET/CT may be a novel marker for evaluating the risk of T2DM and CVD, especially in world’s aging population.

## Methods

Two complementary studies were conducted; (1) a cross-sectional study to assess the relation between amygdalar metabolic activity and clinical variables, and (2) a single-center study involving 16 weeks of study-drug administration and follow-up.

### Subjects and study design 1

The study involved 346 consecutive subjects who underwent a risk-screening test for CVD in Kurume University Hospital. We excluded any patients with history of malignancies, uncontrolled diabetes (FPG ≥ 200 mg/dL), insulin treatment, acute infections, and active inflammatory diseases. All participants gave informed consent to participate in this study. The Ethical Committee for the Clinical Research of Kurume University approved this study (approval number: 23121).

### Data collection

Presence of current medication, medical history including smoking habit, and history of cardiovascular disease were assessed by a questionnaire. Waist circumference was measured as an index of the presence or absence of central obesity. Blood pressure was measured in the sitting position using an upright standard sphygmomanometer. Vigorous physical activity and smoking were avoided for at least 30 min before blood pressure and resting heart rate measurements. Blood samples for laboratory assays were obtained following overnight fasting from the antecubital vein in the morning for determinations of aspartate transaminase, alanine transaminase, γ-GTP, LDL cholesterol, HDL cholesterol, triglycerides, FPG, fasting IRI, HbA_1c_, uric acid, eGFR, hsCRP, adiponectin, ADMA, and PEDF. These blood chemistry variables were measured by standard methods at a commercial laboratory (The Kyodo Igaku Laboratory, Fukuoka, Japan and SRL Inc., Tokyo, Japan) as described previously^41^. Serum level of PEDF was determined with an enzyme-linked immunosorbent assay system as described previously^26^. The value for HbA_1c_ (%) is estimated as a National Glycohemoglobin Standardization Program equivalent value (%) calculated by the formula HbA_1c_ (National Glycohemoglobin Standardization Program) (%) = 1.02 × HbA_1c_ (Japan Diabetes Society) (%) + 0.25%^42^. eGFR was calculated using the Modification of Diet in Renal Disease study equation modified with a Japanese coefficient^43^. Hypertension was defined as blood pressure ≥ 140/90 mmHg or current treatment with antihypertensive medication. Diabetes was defined as FPG ≥ 126 mg/dL and/or current treatment with OHAs.

### FDG-PET and CT

FDG-PET combined with CT was performed as described previously^41^. In brief, after at least 12 h-fasting prior to PET scanning, patients received an intravenous injection administration of FDG {4.2 MBq (0.12 mCi)/kg body weight} via the antecubital vein. One hour after the FDG injection, 3-dimensional whole-body PET and CT scans were carried out using an integrated full-ring PET/CT scanner (Gemini-GXL 16; Philips Medical Systems, Inc., Cleveland, Ohio, USA). The subjects rested for 60 min in a comfortable position in a quiet room and were then conveyed to the scanning suite. CT data were used for attenuation correction and lesion localization. After the transmission and emission images were obtained, the images were reconstructed using the 3D line-of-response row-action maximum likelihood algorithm (3D-LOR-RAMLA; Philips, Eindhoven, The Netherlands). Regional FDG activity in the brain was evaluated by the maximum pixel activity value using FineSRT (PDRadiopharma Inc.), an automated brain analysis program, which allows objective assessment in each region of interest for the whole brain. Amygdalar metabolic activity was determined by averaged FDG activity within the right and left amygdalae, and was corrected for mean temporal lobe FDG activity^12–16^. Two blinded cardiologists measured the amygdalar metabolic activity values. The intra-observer or inter-observer variability of regional FDG activity measurements was less than 5%.

### Areas in the abdominal adipose tissue

Areas in the VAT and SAT were estimated by calculating VAT and SAT areas using a standardized method with CT scan and Fat Scan software (N2 System Corp, Osaka, Japan). Detailed information on the methods of measurement of VAT and SAT areas and activities was previously published^44^. In brief, a region of interest of the fat layer was defined by tracing its contour on each scan, and the attenuation range of CT numbers in Hounsfield units for adipose tissue was calculated. The pixels with attenuation values in the selected attenuation range were depicted. From the regions, the total adipose tissue area was calculated by counting the number of pixels in each; the VAT area was subtracted, and the remainder was defined as the SAT area. The adipose tissue area was determined as the average of areas at the umbilical level and the additional 10 levels separated by 4 mm in length in top and bottom from the umbilical level obtained from consecutive CT images^44^. The investigators who performed the measurements of adipose tissue were blinded to the patients’ characteristics.

### Subjects and study design 2

Among total 346 subjects, 36 patients with T2DM (27 males and 9 females, mean age 68.3 ± 7.9 years) were enrolled in the study design 2. OHAs was added to achieve better glycemic control for these T2DM patients. Nineteen (52.8%) of the 36 patients had already received OHAs, including α-glucosidase inhibitors, glinides, glimepiride, and/or metformin. The initial dose of study drugs was based on FPG levels, and then their doses were titrated to obtain target glycemic control defined as a FPG level of 110 mg/dL or lower. During the study period, the patients were instructed not to change their life styles and to continue taking the same dose of any concomitant drugs. At baseline and 16 weeks after additional treatment with the OHAs, amygdalar metabolic activity was re-evaluated by FDG-PET/CT. We excluded any patients with uncontrolled diabetes (FPG ≥ 200 mg/dL), insulin treatment, heart failure (New York Heart Association functional class ≥ II), malignancies, acute infections, and active inflammatory diseases. In the 36 patients with T2DM, we examined the correlation of changes of amygdalar metabolic activity after 16-week additional treatment with OHAs (Δamygdalar metabolic activity, meaning a value of “after”-“baseline”) with Δclinical variables. This study was a sub-analysis of trial to examine the effects of OHAs on vascular inflammation in patients with T2DM^45^. The Ethical Committee for the Clinical Research of Kurume University approved this study (approval number: 06103). All subjects provided written informed consent.

### Statistics

Data were presented as mean values ± standard deviation or medians with the interquartile range. We performed the Shapiro-Wilk test to evaluate the assumption of normality. Statistical analysis was performed by means of appropriate parametric and nonparametric methods. Univariate correlation between amygdalar FDG activity and each variable was analyzed. To determine the independent parameters associated with amygdalar FDG activity, multiple stepwise regression analysis was performed. The LASSO regression analysis was performed as a shrinkage and variable selection method. Pearson’s product-moment correlation test was performed to determine the association between ∆amygdalar FDG activity and ∆clinical variables. P values of less than 0.05 were considered to be statistically significant. All statistical analyses were performed with the use of the SPSS system (SPSS Inc., Chicago, IL, USA).

## Supplementary information

!R1 Supplementary Tables
