# Supplementary material for: Clinical and biochemical factors associated with amygdalar metabolic activity
Source: NPJ Aging. 2025 Jan 25;11(1):2. doi: 10.1038/s41514-025-00194-4 (PMC11762304; doi:10.1038/s41514-025-00194-4)
Supplement: Supplementary file 1 — !R1 Supplementary Tables [file 41514_2025_194_MOESM1_ESM.pdf]

Supplementary Table 1 | Clinical variables in T2DM and non-DM

Clinical variables	T2DM	Non-DM	P value
Number	90	256	
Male, n (%)	70 (77.8)	162 (63.3)	**0.012**
Age, years	66.0±8.3	62.2±9.4	**<0.001**
Body mass index, kg/m^2	24.2±3.4	23.7±3.3	0.195
Waist circumference, cm	88.4±9.4	86.3±9.6	0.080
Heart rate, beats per minute	64.5±9.9	64.0±10.1	0.730
Systolic blood pressure, mmHg	132.9±17.3	136.1±18.4	0.159
Diastolic blood pressure, mmHg	76.4±11.2	81.7±11.1	**<0.001**
Aspartate transaminase*, U/L	24.0 (20.0-30.0)	22.0 (19.0-27.0)	0.126
Alanine transaminase*, U/L	22.5 (18.0-32.3)	19.0 (15.0-27.0)	0.011
γ-glutamyl transferase*, U/L	32.0 (22.0-63.5)	28.0 (19.0-51.8)	0.140
LDL cholesterol, mg/dL	119.8±30.0	124.9±29.1	0.156
HDL cholesterol, mg/dL	50.9±12.9	59.3±13.8	**<0.001**
Triglycerides*, mg/dL	115.0 (72.0-164.0)	99.5 (74.0-143.8)	0.112
Fasting plasma glucose*, mg/dL	129.5 (118.0-144.3)	97.0 (91.0-103.0)	**<0.001**
Fasting immunoreactive insulin*, µU/mL	6.20 (3.78-9.13)	4.75 (3.40-7.60)	**0.007**
Glycated hemoglobin, %	6.88±0.94	5.75±0.33	**<0.001**
Uric acid, mg/dL	5.93±1.39	5.65±1.38	0.102
Estimated glomerular filtration rate, mL/min	72.4±19.1	78.8±17.3	**0.004**
High-sensitivity CRP*, mg/L	0.63 (0.27-1.45)	0.47 (0.23-0.92)	0.117
ADMA*, nmoL/mL	0.52 (0.46-0.59)	0.46 (0.42-0.51)	**<0.001**
PEDF*, mg/mL	12.70 (9.31-17.53)	11.80 (8.97-15.78)	0.277
Visceral adipose tissue area, cm^2	121.1±45.8	117.9±45.4	0.611

Subcutaneous adipose tissue area, cm^2	131.9±51.4	126.2±36.1	0.319
Amygdalar metabolic activity	0.75±0.06	0.71±0.06	**<0.001**
Active smoker, n (%)	15 (16.7)	48 (18.8)	0.660
Cerebrovascular disease, n (%)	8 (8.9)	3 (1.2)	**0.001**
Coronary artery disease, n (%)	25 (27.8)	8 (3.1)	**<0.001**
Drugs, n (%)			
Aspirin	33 (36.7)	19 (7.4)	**<0.001**
Statins	36 (40.0)	53 (20.7)	**<0.001**
Anti-hypertensive agents	58 (64.4)	79 (30.9)	**<0.001**
Oral hypoglycemic agents	48 (53.3)	0 (0.0)	**<0.001**
Sleeping pills	7 (7.8)	19 (7.4)	0.912

Data are presented as number (%), mean ± SD, or *median (interquartile range). LDL, low-density lipoprotein; HDL, high-density protein; hsCRP, high-sensitivity C-reactive protein; ADMA, asymmetric dimethyl arginine; PEDF, pigment epithelium-derived factor.

Supplementary Table 2 | Clinical variables at baseline and after additional OHAs therapy in the study 2

Parameters	Baseline	Post treatment	P value
Number	36	36	
Male, n (%)	27 (75.0)	27 (75.0)	
Age, years	68.3±7.9	68.6±7.9	
Weight, kg	65.0±11.8	65.8±11.2	**0.011**
Waist circumference, cm	88.9±10.1	89.8±9.5	**0.036**
Heart rate, beats per minute	62.2±8.3	63.2±8.7	0.478
Systolic blood pressure, mmHg	127.9±14.2	126.3±14.8	0.412
Diastolic blood pressure, mmHg	70.1±9.8	69.4±10.0	0.554
Aspartate transaminase*, U/L	24.5 (21.0-29.0)	23.5 (19.3-30.8)	0.293
Alanine transaminase*, U/L	22.0 (18.0-32.5)	20.5 (16.0-29.5)	0.054
γ-glutamyl transferase*, U/L	30.5 (20.0-48.3)	29.0 (16.0-45.0)	0.062
LDL cholesterol, mg/dL	116.6±24.9	115.8±21.8	0.830
HDL cholesterol, mg/dL	50.4±13.7	53.7±13.9	**0.009**
Triglycerides*, mg/dL	118.0 (73.0-142.5)	118.0 (82.3-182.5)	**0.031**
Fasting plasma glucose*, mg/dL	130.5 (118.5-142.3)	117.5 (110.0-127.0)	**<0.001**
Fasting immunoreactive insulin*, µU/mL	6.30 (3.80-10.78)	6.25 (4.25-8.50)	0.110
Glycated hemoglobin, %	6.88±0.60	6.44±0.44	**<0.001**
Uric acid, mg/dL	6.19±1.73	6.36±1.73	0.311
Estimated glomerular filtration rate, mL/min	66.5±17.5	66.8±17.3	0.784
High-sensitivity CRP*, mg/L	0.74 (0.32-1.80)	0.61 (0.32-1.39)	0.518
ADMA*, nmoL/mL	0.60 (0.53-0.68)	0.54 (0.51-0.61)	**0.001**
PEDF*, mg/mL	14.00 (10.90-21.00)	14.80 (12.10-18.00)	0.571
Visceral adipose tissue area, cm^2	116.8±46.4	114.1±44.6	0.435
Subcutaneous adipose tissue area, cm^2	137.6±57.1	141.1±57.6	0.382

Amygdalar metabolic activity	0.74±0.06	0.74±0.05	0.523
Active smoker, n (%)	7 (19.4)	7 (19.4)	
Cerebrovascular disease, n (%)	8 (22.2)	8 (22.2)	
Coronary artery disease, n (%)	19 (52.8)	19 (52.8)	
Drugs, n (%)			
Aspirin	24 (66.7)	24 (66.7)	
Statins	19 (52.8)	19 (52.8)	
Anti-hypertensive agents	34 (94.4)	34 (94.4)	
Oral hypoglycemic agents	19 (52.8)	36 (100.0)	
Sleeping pills	0 (0.0)	0 (0.0)	

Abbreviations as in Supplementary Table 1.

Supplementary Table 3 | Associations between baseline clinical variables and Δamygdalar metabolic activity

Parameters	Univariate	
	β	P value
Gender†	-0.096	0.576
Age	-0.033	0.848
Body weight	0.056	0.746
Waist circumference	0.040	0.817
Body mass index	0.003	0.987
Heart rate	-0.023	0.893
Systolic blood pressure	-0.210	0.218
Diastolic blood pressure	0.101	0.556
Aspartate transaminase*	-0.147	0.394
Alanine transaminase*	-0.155	0.366
γ-glutamyl transferase*	-0.136	0.428
LDL cholesterol	0.202	0.238
HDL cholesterol	0.252	0.138
Triglycerides*	-0.197	0.250
Fasting plasma glucose*	-0.223	0.191
Fasting immunoreactive insulin*	-0.140	0.415
Glycated hemoglobin	-0.301	0.074
Estimated glomerular filtration rate	0.055	0.749
Uric acid	-0.239	0.160
High-sensitivity CRP*	-0.150	0.383
ADMA*	-0.270	0.111
PEDF*	-0.603	**<0.001**
Visceral adipose tissue area	0.087	0.612
Subcutaneous adipose tissue area	-0.066	0.702

† Men=0, Women=1. * Log-transformed value was used.

β: regression coefficients.

Abbreviations as in Supplementary Table 1.

Supplementary Table 4 | Association of Δclinical variables with Δamygdalar metabolic activity excluding the data of ΔPEDF

Parameters	Univariate		Multivariate	
	β	P value	β	P value
ΔBody weight	-0.174	0.309	-	-
ΔWaist circumference	-0.226	0.185	-	-
ΔHeart rate	-0.081	0.638	-	-
ΔSystolic blood pressure	0.153	0.372	-	-
ΔDiastolic blood pressure	-0.086	0.619	-	-
ΔAspartate transaminase*	0.142	0.408	-	-
ΔAlanine transaminase*	0.323	0.055	-	-
Δγ-glutamyl transferase*	0.372	**0.025**	0.266	0.105
ΔLDL cholesterol	0.032	0.853	-	-
ΔHDL cholesterol	-0.186	0.277	-	-
ΔTriglycerides*	-0.058	0.735	-	-
ΔFasting plasma glucose*	0.206	0.229	-	-
ΔFasting immunoreactive insulin*	-0.099	0.565	-	-
ΔGlycated hemoglobin	0.060	0.728	-	-
ΔUric acid	0.277	0.102	-	-
ΔHigh-sensitivity CRP*	0.332	**0.048**	0.232	0.154
ΔADMA*	0.423	**0.010**	0.423	**0.010**
ΔPEDF*	-	-	-	-
ΔVisceral adipose tissue area	0.001	0.993	-	-
ΔSubcutaneous adipose tissue area	-0.226	0.185	-	-
R^2			0.155	

* Log-transformed value was used.

β: regression coefficients.

Abbreviations as in Supplementary Table 1.

Supplementary Table 5 | Clinical variables stratified by median value of ∆amygdalar metabolic activity

Parameters	High ∆amygdalar metabolic activity group (≤ -0.013)	Low ∆amygdalar metabolic activity group (> -0.013)	P value
Number	18	18	
Age, years	69.7±7.5	66.9±8.2	0.298
Male, number	12	15	0.441
Amygdalar metabolic activity			
Baseline	0.77±0.04	0.72±0.07	**<0.001**
Post treatment	0.72±0.04	0.75±0.05	0.069
P value vs baseline	**0.012**	0.089	
Weight, kg			
Baseline	64.1±11.3	66.0±12.3	0.644
Post treatment	65.0±10.7	66.6±11.7	0.679
P value vs baseline	0.054	0.111	
Waist circumference, cm			
Baseline	89.2±7.2	88.6±12.4	0.872
Post treatment	90.3±7.0	89.3±11.4	0.751
P value vs baseline	0.059	0.298	
Heart rate, beats per minute			
Baseline	60.6±7.3	63.9±8.8	0.239
Post treatment	61.8±9.2	64.5±8.0	0.372
P value vs baseline	0.483	0.763	
Systolic blood pressure, mmHg			
Baseline	128.0±12.1	127.8±16.1	0.964
Post treatment	124.9±13.8	127.7±15.7	0.580
P value vs baseline	0.253	0.984	
Diastolic blood pressure, mmHg			
Baseline	69.0±8.1	71.3±11.1	0.498
Post treatment	68.9±8.1	69.9±11.7	0.773
P value vs baseline	0.959	0.330	

Aspartate transaminase*, U/L			
Baseline	27.0 (20.8-36.8)	22.5 (20.5-28.3)	0.222
Post treatment	26.0 (18.5-34.0)	23.5 (19.8-27.3)	0.555
P value vs baseline	0.153	0.977	
Alanine transaminase*, U/L			
Baseline	26.0 (19.0-36.0)	20.5 (16.3-26.3)	0.182
Post treatment	21.5 (15.3-27.8)	19.5 (15.8-34.3)	0.913
P value vs baseline	**0.004**	0.983	
γ-glutamyl transferase*, U/L			
Baseline	37.0 (20.0-69.5)	26.5 (18.3-42.3)	0.172
Post treatment	29.5 (16.0-46.3)	28.0 (16.0-44.3)	0.746
P value vs baseline	**0.004**	0.712	
LDL cholesterol, mg/dL			
Baseline	115.6±23.9	117.5±25.8	0.818
Post treatment	113.2±23.3	118.3±19.9	0.498
P value vs baseline	0.546	0.898	
HDL cholesterol, mg/dL			
Baseline	45.8±10.1	55.0±15.2	**0.046**
Post treatment	50.1±9.2	57.2±16.7	0.132
P value vs baseline	**0.027**	0.177	
Triglycerides*, mg/dL			
Baseline	124.5 (90.8-176.0)	92.0 (66.0-126.5)	**0.009**
Post treatment	126.5 (107.0-210.3)	99.5 (78.8-148.5)	**0.042**
P value vs baseline	0.265	0.054	
Fasting plasma glucose*, mg/dL			
Baseline	136.0 (117.8-161.0)	127.0 (120.0-134.8)	0.250
Post treatment	117.5 (108.0-129.0)	117.0 (110.8-127.5)	0.745
P value vs baseline	**0.002**	**<0.001**	
Fasting immunoreactive insulin*, μU/mL			

Baseline	6.10 (3.78-10.90)	6.30 (3.98-9.80)	0.632
Post treatment	7.05 (4.15-9.20)	5.15 (4.05-7.58)	0.352
P value vs baseline	0.557	**0.029**	
Glycated hemoglobin, %			
Baseline	7.00±0.66	6.76±0.50	0.243
Post treatment	6.49±0.49	6.38±0.39	0.467
P value vs baseline	**<0.001**	**<0.001**	
High-sensitivity CRP*, mg/L			
Baseline	0.85 (0.44-2.07)	0.58 (0.28-1.27)	0.175
Post treatment	0.65 (0.31-1.31)	0.61 (0.37-1.85)	0.475
P value vs baseline	**0.004**	0.212	
ADMA*, nmoL/mL			
Baseline	0.60 (0.55-0.69)	0.63 (0.53-0.66)	0.821
Post treatment	0.53 (0.49-0.60)	0.54 (0.51-0.62)	0.489
P value vs baseline	**0.009**	0.052	
PEDF*, mg/mL			
Baseline	17.10 (11.55-23.85)	12.80 (8.05-18.00)	0.069
Post treatment	15.65 (12.05-17.63)	14.00 (10.80-18.75)	0.447
P value vs baseline	0.431	0.163	
Visceral adipose tissue area, cm^2			
Baseline	114.7±36.9	118.8±54.2	0.799
Post treatment	114.9±41.0	113.2±48.0	0.910
P value vs baseline	0.970	0.184	
Subcutaneous adipose tissue area, cm^2			
Baseline	141.7±45.4	133.4±66.6	0.673
Post treatment	155.7±56.2	126.5±55.3	0.136
P value vs baseline	**0.015**	0.199	

Abbreviations as in Supplementary Table 1.
